# Effects of Orexin B on Swine Granulosa and Endothelial Cells

**DOI:** 10.3390/ani11061812

**Published:** 2021-06-17

**Authors:** Francesca Grasselli, Simona Bussolati, Stefano Grolli, Rosanna Di Lecce, Cecilia Dall’Aglio, Giuseppina Basini

**Affiliations:** 1 Dipartimento di Scienze Medico-Veterinarie, Università degli Studi di Parma, Via del Taglio 10, 43126 Parma, Italy; francesca.grasselli@unipr.it (F.G.); simona.bussolati@unipr.it (S.B.); stefano.grolli@unipr.it (S.G.); rosanna.dilecce@unipr.it (R.D.L.); 2 Dipartimento di Medicina Veterinaria, Università degli Studi di Perugia, Via San Costanzo 4, 06126 Perugia, Italy; cecilia.dallaglio@unipg.it

**Keywords:** ovarian follicle, oxidative stress, estradiol 17 β, progesterone, angiogenesis

## Abstract

**Simple Summary:**

The follicle is the ovarian functional unit. It is mainly composed of granulosa cells and angiogenesis is crucial to guarantee its development till ovulation. Carrying on our previous studies on the orexin system in the ovary, we presently demonstrate a potential role of orexin B in the control of granulosa cells’ oxidative stress and of the angiogenesis event.

**Abstract:**

In addition to the well-known central modulatory role of orexins, we recently demonstrated a peripheral involvement in swine granulosa cells for orexin A and in adipose tissue for orexin B (OXB). The aim of present research was to verify immunolocalization of OXB and its potential role in modulating the main features of swine granulosa cells. In particular, we explored the effects on granulosa cell proliferation (through the incorporation of bromodeoxyuridine), cell metabolic activity (as indirect evaluation by the assessment of ATP), steroidogenic activity (by immunoenzymatic examination) and redox status (evaluating the production of superoxide anion by means of the WST test, production of nitric oxide through the use of the Griess test and the non-enzymatic reducing power by FRAP test). Our data point out that OXB does not modify granulosa cell growth, steroidogenesis and superoxide anion generation. On the contrary, the peptide stimulates (*p* < 0.05) nitric oxide output and non-enzymatic reducing power. Since new vessel growth is crucial for ovarian follicle development, a further aim of this study was to explore the expression of prepro-orexin and the effects of OXB on swine aortic endothelial cells. We found that the peptide is ineffective in modulating cell growth, while it inhibits redox status parameters. In addition, we demonstrated a stimulatory effect on angiogenesis evaluated in fibrin gel angiogenesis assay. Taken together, OXB appears to be potentially involved in the modulation of redox status in granulosa and endothelial cells and we could argue an involvement of the peptide in the follicular angiogenic events.

## 1. Introduction

Since their discovery in 1998 in the lateral hypothalamus [1,2], the neuropeptides orexin A and B and their receptors, OXR1 and OXR2, have been found to be widely distributed both in the nervous system and in many peripheral tissues. Several reports have recently highlighted the involvement of the orexin system in the regulation of many physiological processes, such as food intake and energy homeostasis [3], wakefulness and sleep [4], blood pressure [5] and emotional behaviors [6]. A modulatory effect of orexin B on adipose stromal cells’ viability and adipogenic differentiation in the pig was recently shown, supporting the hypothesis that the orexinergic system could play a role in the physiology of adipose tissue, which is now recognized to possess different endocrine and metabolic functions [7,8,9]. Many indications exist concerning the potential role of orexins in the control of various endocrine axes, among which the hypothalamic–pituitary–gonadal axis is included [10,11,12,13]. Within this perspective, although the role of the orexin system as an integrative link between energy homeostasis and reproduction has been hypothesized, its involvement in the regulation of the female reproductive system needs to be further clarified. The expression of prepro-orexin (PPO) gene and the presence of receptors have been confirmed in the ovary of different species [10,11,14,15,16]. In a recent work [17], we documented that porcine granulosa cells from large follicles express PPO mRNA as well as orexin receptors. Moreover, our findings about the effects of OXA on granulosa cell functional activity indicate that OXA and its receptors could modulate female reproductive functions through the control of ovarian steroidogenesis. In contrast, the role of OXB and OX2R in the regulation of reproductive activity has been poorly investigated. Kaminski et al. [18] and Kisielewska et al. [19] documented an involvement of OXB in the regulation of steroidogenesis by the porcine uterus during the luteal phase and early pregnancy. As for the gonads, Liguori et al. [20] provided the first evidence of OXB and OXR2 localization within the rat epididymis. In the female, a modulatory effect of OXB on ovarian steroidogenic activity was documented by Cataldi et al. [21] in the rat and Nitkiewicz et al. [12] in the pig. On these bases, this study was undertaken to localize OXB in sections of the whole porcine ovary as well as to examine the in vitro effect of different OXB concentrations on cell growth, steroidogenesis and redox status of granulosa cells [16,17]. Moreover, a further aim of the present research was to investigate the possible localization of OXB in follicular blood vessels, as we recently documented for OXA and its receptors [15]. These observations led us to hypothesize an involvement of the orexin system in the control of ovarian angiogenesis, a process tightly related to follicular growth and maturation. Thus, the present research was also aimed at studying this aspect in a swine aortic endothelial cell (AOC) model [22], firstly investigating the expression of the PPO gene. Moreover, the effect of OXB on AOC growth and redox status parameters was taken into account. In addition, the effect of OXB was explored using our previously set up fibrin gel angiogenesis bioassay [23].

## 2. Materials and Methods

All reagents used in this study were obtained from Sigma (St. Louis, MO, USA) unless otherwise specified.

### 2.1. Collection of Ovaries

For each experiment, we collected ovaries at a local slaughterhouse from 40 Large White cross-bred gilts (parity = 0, aged 8–9 mo, weighing about 180 kg), immediately after death. The evaluation of the estrous cycle was carried out on the basis of ovarian morphology as previously described [24,25]. Ovaries were placed into cold PBS (4 °C) supplemented with penicillin (500 IU/mL), streptomycin (500 μg/mL) and amphotericin B (3.75 μg/mL), maintained in a freezer bag and transported to the laboratory within 1 h [9].

### 2.2. Immunolocalization of OXB

The ovaries collected from six gilts were treated with routine preparation of IHC on paraffin-embedded tissues for immunolocalization [17]. Briefly, tissues were fixed in 10% buffered formalin and embedded in paraffin. We obtained sections 5 µm thick which were routinely stained (hematoxylin and eosin) and used for immunohistochemical studies by means of mouse monoclonal antibody anti-orexin B (MAB734, R&D Systems, Inc., Minneapolis, MN, USA) [26]. Negative control sections were incubated with mouse IgG_1_ isotype control (MAB002, R&D Systems, Inc., Minneapolis, MN, USA) using the same concentration of the primary antibody. Antigen retrieval was carried out by dipping the sections in 0.01 M sodium citrate buffer, pH 6.0, and heating them in a microwave oven for 15 min at 400 W. Thereafter, we incubated the sections in: (1) 3% hydrogen peroxidase for 15 min to block endogenous peroxidase; (2) primary antibody anti-orexin B at a dilution of 1:200 for 1 h at room temperature; (3) biotinylated goat anti-mouse IgG (BA-9200 Vector laboratories) at a dilution 1:200 for 30 min; (4) Vectastain Elite ABC Peroxidase Kit (PK-6100) for 30 min; and (5) 3,3-diaminobenzidine (DAB substrate kit, peroxidase—Vector laboratories SK-4100) for 5 min. After each step, washings of the sections were carried out with phosphate-buffered saline, pH 7.0 (PBS). The sections were counterstained with Mayer’s hematoxylin solution. Histological slides were examined with a Nikon Eclipse E800 microscope (Nikon Corporation, Minato-Ku, Japan). Pictures of the sections were obtained with a DIGITAL SIGHT DS-Fi1 camera (Nikon Corporation, Minato-Ku, Japan)

### 2.3. Evaluation of OXB Effect on Swine Granulosa Cell Function

Morphological criteria were used to classify swine ovarian follicles as healthy. Atretic follicles and those with hemorrhagic, opaque or “milky” follicular fluid were excluded [27]. On the basis of our previous studies [17,22,28,29], as well as the classification of Foxcroft and Hunter [30], granulosa cells were aseptically harvested by aspiration of healthy follicles > 5 mm diameter with a 26-gauge needle and released in medium containing heparin (50 IU/mL). In order to also collect mural cells, granulosa cell collection was associated with a gentle scraping of the follicle wall with the needle. Cells were then centrifuged for pelleting and treated with 0.9% prewarmed ammonium chloride at 37 °C for 1 min to remove red blood cells. In order to evaluate cell number and viability we used a hemocytometer under a phase contrast microscope after vital staining with trypan blue (0.4%) of an aliquot of the cell suspension. After collection, cells were seeded in culture medium represented by DMEM/Ham’s F12 supplemented with sodium bicarbonate (2.2 mg/mL), bovine serum albumin (BSA 0.1%), penicillin (100 IU/mL), streptomycin (100 μg/mL), amphotericin B (2.5 μg/mL), selenium (5 ng/mL) and transferrin (5 μg/mL), indicated hereafter as CM. After seeding, cells were incubated for 48 h at 37 °C under a humidified atmosphere (5% CO_2_) with or without OXB at the concentration of 0.1, 1 and 10 nM, previously examined in another study [12] and demonstrably close to the physiological range in swine blood [31].

#### 2.3.1. Granulosa Cell Viability and Proliferation

We studied cell viability by means of MTT test (Sigma, St. Louis, MO, USA), which is a colorimetric assay based on tetrazolium salt 3-[4,5-dimethylthrazol-2-yl]-2,5-dipheniltetrazolium bromide (MTT) reduction by mitochondrial dehydrogenase [32]. A total of 10^4^ cells were seeded in 96-well plates in 200 μL CM and treated with OXB for 48 h, as indicated above. After each incubation, we added MTT (5 mg/mL) to cultured cells which were then incubated for 4 h. In order to solubilize the reduction product, formazan, we added 100 µL of lysis solution (SDS 10% in HCl 0.01 N) to each well and left them at 37 °C overnight. The absorbance was measured at 540 nm with Victor Reader (Perkin Elmer, Groningen, The Netherlands)

We determined cell proliferation with the ELISA BrdU (Roche Diagnostic, Indianapolis, IN, USA), an immunological colorimetric assay used for quantitative analysis, since BrdU can be incorporated into the newly synthesized DNA of replicating cells. Granulosa cells were plated into 96-well plates (Sarstedt, Nümbrecht, Germany) (10^4^ cells/200 µL of CM), and they were treated with the substance of interest and incubated overnight at 37 °C in a humidified atmosphere [33]. Thereafter, they were incubated with 20 μL of BrdU for 24 h. After incubation, cells were fixed and DNA was denatured before adding the anti-BrdU antibody, conjugated with the enzyme peroxidase. During the 2 h-incubation, we added 100 μL of tetramethylbenzidine substrate (TMB), which develops a blue color after being oxidized by the enzyme in a quantity proportional to the amount of newly synthesized DNA. At the end, we stopped the reaction by adding 25 µL of sulfuric acid (1M) which caused the sample to turn yellow. We measured absorbance values at 450 nm using a Victor Reader spectrophotometer. In order to determine viable cell number, the absorbance of each sample was read against a standard curve prepared by culturing, in quintuplicate, granulosa cells at different plating densities (from 10^3^ to 10^5^ viable/200 μL) for 48 h. The curve was repeated in four different experiments. The relationship between cell number and absorbance was linear (*r* = 0.92). Cell number/well was estimated from the resulting linear regression equation and was used to correct experimental data. The assay detection limit was 10^3^ cell/well and the variation coefficient was less than 5%.

#### 2.3.2. Granulosa Cell Steroid Production

A total of 10^4^ viable cells/well were seeded in 96-well plates in 200 μL CM supplemented with 28 ng/mL androstenedione [29] and treated with OXB for 48 h as above indicated. Thereafter, we collected culture media which were frozen and stored at −20 °C until progesterone (P4) and estradiol 17β (E2) determination by means of direct immunoenzymatic determination (Dia.Metra SRL, Spello, PG, Italy) [34]. The kits are based on competitive colorimetric immunoassay methods. As for the Estradiol ELISA kit, sample media were incubated at 37 °C for 2 h and, after three washings, we added 100 µL of TMB substrate; its reaction with H_2_O_2_ was catalyzed by the HPR enzyme present in the bound fraction. After a 30 min incubation in the dark the reaction product develops a blue color that turns to yellow after the addition of the stop solution. The concentration of the hormone is determined on the basis of a 5-point calibration curve from 0 to 2000 pg/mL. The data are processed by the spectrophotometer; the absorbance is read at 450 nm against a reference wavelength of 620–630 nm. The within-assay variability was < 9%. The direct immunoenzymatic determination of progesterone was performed on the same bases; the concentration of progesterone in the sample was calculated against a 4-point calibration curve from 0 to 40.0 ng/mL. The ELISA progesterone kit requires a 1 h incubation at 37 °C; after the removal of the unbound antibody, we added 100 μL of substrate TMB and we left the plate to incubate for 15 min at 37 °C away from light. After the stopping of reaction, the absorbance was read at 450 nm against a reference wavelength of 620–630 nm using the Victor Reader. The within-assay variability was <4%.

#### 2.3.3. Granulosa Cell Redox Status

##### Granulosa Cell Superoxide (O_2_^−^) Production

O_2_^−^ production was evaluated by WST-1-(4-[3-(4-iodophenyl)-2-(4-nitrophenyl)-2H-5-tetrazolium]-1,3-benzene disulfonate) test (Roche, Mannheim, Germany). The assay is based on the cleavage of the water-soluble tetrazolium salt WST-1 to a yellow–orange, water-soluble formazan. Experimental data document that tetrazolium salts can be used as a reliable measure of intracellular O_2_^−^ production [22]. A total of 10^4^ viable cells/200 µL CM were seeded in 96-well plates, treated with OXB as above described and incubated for 48 h. During the last 4 h of treatment, we added 20 μL WST-1 to cells and absorbance was then determined using the Victor Reader at a wavelength of 450 against 620 nm. 

##### Granulosa Cell Nitric Oxide (NO) Production

A total of 10^5^ viable cells/200 μL CM were seeded in 96-well plates and treated with OXB for 48 h as previously described. NO was quantified by measuring nitrite levels in culture media by the microplate method based on the formation of chromophores after reaction with Griess reagent, which was prepared fresh daily by mixing equal volumes of stock A (1% sulfanilamide, 5% phosphoric acid) and stock B (0.1% *N*-[naphthyl] ethylenediamine dihydrochloride) [35].

##### Granulosa Cell Non-Enzymatic Scavenging Activity

The ferric reducing ability of plasma (FRAP) assay is a colorimetric method based on the ability of the antioxidant molecules to reduce ferric-tripiridyltriazine (Fe^3+^ TPTZ) to a ferrous form (Fe^2+^ TPTZ). Fe^2+^ is measured spectrophotometrically via determination of its colored complex with 2,4,6-Tris(2-pyridyl)-s-triazine (Fe^2+^ TPTZ). TPTZ reagent was prepared before use, mixing 25 mL of acetate buffer, 2.5 mL of 2,4,6-Tris(2-pyridyl)-s-triazine (TPTZ) 10 mM in HCl 40 mM and FeCl^3−^ 6H_2_O solution. Briefly, 2 × 10^5^ viable cells/200 μL CM were seeded in 96-well plates and treated with OXB for 48 h as previously described. At the end, plates were centrifuged for 10 min at 400× *g*, supernatants were discarded and cells were lysed by adding cold Triton 0.5% + PMSF in PBS (200 μL/well), incubating on ice for 30 min. The test was carried out on 40 μL of cell lysates added to Fe^3+^ TPTZ reagent and then incubated at 37 °C for 30 min. The absorbance of Fe^2+^ TPTZ was determined by Victor Reader at 595 nm. We determined the ferric reducing ability of cell lysates by plotting a standard curve of absorbance against FeSO_4_^−^ 7H_2_O standard solution [36].

### 2.4. Evaluation of OXB Effects on Swine Aortic Endothelial Cell (AOC) Function

#### 2.4.1. AOC Culture

The immortalized porcine aortic endothelial cell line (AOC) used in the experiments was kindly provided by Prof. Jose Yélamos (Hospital Universitario Virgen de la Arrixaca, El Palmar, Murcia, Spain) [27]. We used cells at the 13th passage which were grown in Medium 199 (containing Earle’s salts and l-glutamine) supplemented with sodium bicarbonate (2.2 mg/mL), penicillin (100 IU/mL), streptomycin (100 µg/mL), amphotericin B (2.5 mg/mL) and 20% FBS (fetal bovine serum) (GIBCO^TM^, Invitrogen Corporation, Renfrew, UK), indicated as CM2, and incubated at 37 °C in a humidified atmosphere (5% CO_2_).

#### 2.4.2. AOC Prepro-Orexin Expression Evaluation

Total RNA was extracted from 10^6^ AOCs using Nucleospin® RNA II (Macherey-Nagel Gmbh, Duren, Germany) according to the manufacturer’s instructions. Total RNA was quantified by absorbance at 260 nm (Gen Quant Pro, Amersham Bioscences, Freiburg, Germany). Total RNA (2 μg) was reverse transcribed with RevertAid First Strand cDNA Synthesis (Thermo Fisher Scientific, Waltham, MA, USA). Semiquantitative analysis of prepro-orexin (PPO) gene expression (accession number EF434655) was performed by nested PCR. We carried out two sequential runs of PCR with two different sets of primers complementary to swine PPO cDNA (nested PCR). The primers used for the second run were designed to amplify a sequence target within the first run product. For the first amplification run, the following primer pair was used: forward 5′-AAGACGACACCCTTCCTGGAGAC-3′; reverse 5′-TGATTGCCAGCGCCGTGTAGCA-3′ (amplicon length: 221 bp). After a first 1 min denaturation step at 94 °C, the following PCR cycle was repeated 35 times: 94 °C for 30 s, then 58 °C for 30 s and 72 °C for 30 s. A final elongation step was performed at 72 °C for 10 min. For the second PCR run, the following nested primers were used: forward 5′-TCCTTTTCGAAAGGTCTCCTGG-3′, and reverse 5′-GTGTAGCAGCTCGTAGAGGCG-3′ (amplicon length: 154 bp). The amplification was performed at the same conditions as described above except for cycle number (34). Actin (ACT) was chosen as housekeeping gene co-amplified with the interest genes [17,32,37]. 

#### 2.4.3. AOC Viability and Proliferation

In order to evaluate cell viability and cell proliferation, we seeded AOC in 96-well plates at a density of 1.5 × 10^3^ cells/well in 200 μL CM2 and incubated them at 37 °C in a humidified atmosphere (5% CO_2_). After 24 h, medium was changed and additional incubation was performed with or without OXB 10 nM for 48 h. Cell viability was evaluated by MTT assay and cell proliferation was measured with BrdU test as above described for granulosa cells.

#### 2.4.4. AOC Redox Status

A total of 1.5 × 10^3^ viable cells/200 μL CM2 were seeded in 96-well plates and incubated at 37 °C in a humidified atmosphere (5% CO_2_). After 24 h, medium was renewed and cells were treated for 48 h with OXB 10 nM. WST-1 was added to cells during the last 4 h to quantify O_2_^−^ output as previously described for granulosa cells.

A total of 5 × 10^4^ viable cells/200 μL CM2 were seeded in 96-well plates and incubated at 37 °C in a humidified atmosphere (5% CO_2_ and 19% O_2_). After 24 h, medium was renewed and cells were treated for 48 h with OXB 10 nM. At the end of the incubation period, the media were collected, and NO content was assayed with Griess reagents, as described above for granulosa cells.

A total of 5 × 10^4^ viable cells/200 μL CM2 were seeded in 24-well plates and incubated at 37 °C in a humidified atmosphere (5% CO_2_ and 19% O_2_) for 24 h. The medium was then renewed, and the cells were incubated for 48 h with OXB 10 nM. Then, cells were lysed and non-enzymatic scavenger activity was assayed with the FRAP method, as previously described for granulosa cells.

#### 2.4.5. Angiogenesis Bioassay

The microcarrier-based fibrin gel angiogenesis assay was carried out as described [38] with some modifications. Briefly, 12.5 mg gelatin-coated cytodex-3 microcarriers in 1 mL PBS were incubated for 3 h to hydrate. After two washings in PBS and one in CM2, the microcarriers were put in flasks containing 5 mL CM2; AOC (5 × 10^5^) were added and cultured for 24 h in order to let the endothelial cells coat the microcarriers. For the fibrin gel preparation, 40 μL microcarriers covered by AOC were pipetted into 6-well plates containing a solution of fibrinogen (1 mg/mL PBS, pH 7.6), with 1250 IU thrombin (250 μL). Fibrin gels were allowed to polymerize for 30 min at 37 °C, and then were equilibrated for 60 min with 2 mL M199. After medium renewal, we incubated AOC with or without OXB 10 nM. Plates were incubated at 37 °C under a humidified atmosphere (5% CO_2_). AOC were cultured for 72 h, and the treatment was completely renewed after 48 h as described above. Endothelial cell proliferation in the fibrin gel matrix was evaluated by means of the public domain NIH Program Scion Image Beta 4.02. Ten pictures were taken for each gel at 48 and 72 h; images were converted into gray scale, resized to 50% (Paintbrush Software, MS Office) and saved in a Bitmap 24 bit format compatible with Scion. The modified images were then imported into the program and measurements were carried out by drawing the perimeter of the area occupied by AOC expressed as number of pixels. In order to validate the measurement of the area covered by AOC in fibrin gels as a reliable method to evaluate cell proliferation, we stained fibrin gels with the nuclear dye bisbenzimide (Hoechst 33258, 20 μg/mL in PBS for 60 min) and then we examined them by fluorescence microscope. This procedure was performed 20 times; for each experiment the number of nuclei was counted under fluorescence and pictures of the area covered by AOC were taken in order to measure the surface covered in the fibrin gel. A strong correlation was observed between the area covered by AOC and the number of nuclei found in the same area (*r* = 0.96).

### 2.5. Statistical Analysis

The experiments were repeated at least 5 times (6 replicates/treatment). Every time the ovaries were collected from 40 gilts. Experimental data are presented as mean ± SEM; statistical differences among treatments were calculated with ANOVA using Statgraphics package (STSC Inc., Rockville, MD, USA). When significant differences were found, means were compared by Scheffè’s F test; *p* values < 0.05 were considered to be statistically significant.

## 3. Results

### 3.1. Immunolocalization of OXB and Its Effects on Swine Granulosa Cell Function

**Figure 1:**
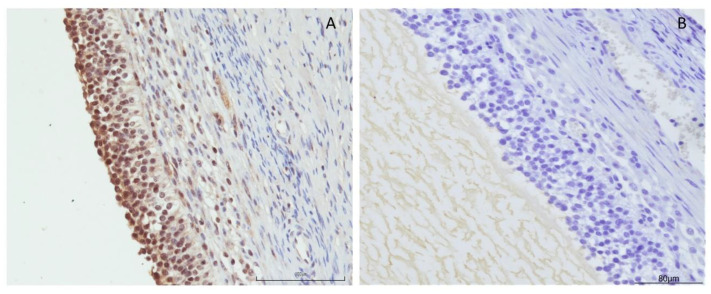
(**A**) Cytoplasmatic positivity for orexin B in granulosa cells (20X IHC). Scale bar = 100 µm. (**B**) Negative control.

Immunohistochemical cytoplasmatic positivity was observed for OXB both in granulosa and in thecal cells of swine ovarian follicles (Figure 1).

**Figure 2:**
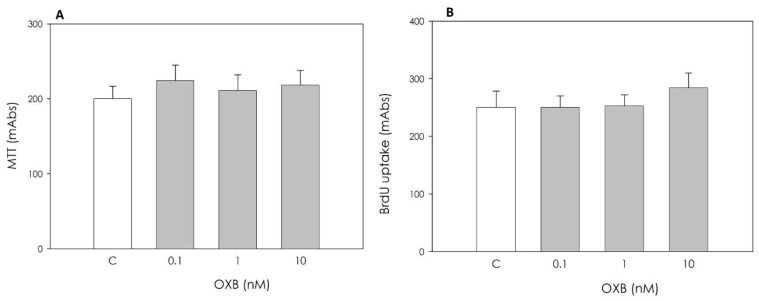
Effect of the 48 h treatment with orexin B (OXB; 0.1, 1 and 10 nM) on swine granulosa cell viability, evaluated by MTT test (**A**), and on granulosa cell proliferation evaluated using 5-bromo-2′-deoxyuridine (BrdU) incorporation assay test (**B**). Data, expressed as milliAbsorbance (mAbs), represent the mean ± SEM of six replicates/treatment repeated in five different experiments.

OXB did not affect either granulosa cell viability or granulosa cell proliferation at the concentrations tested (Figure 2).

**Figure 3:**
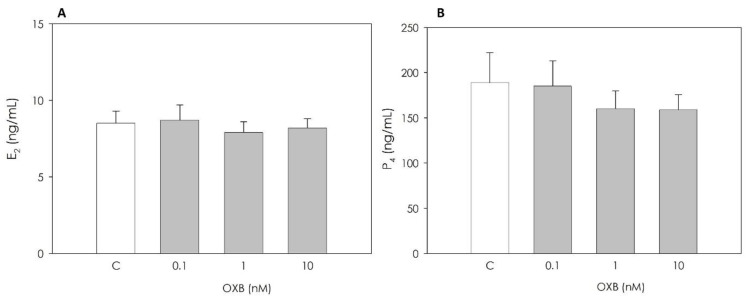
Effect of the 48 h treatment with orexin B (OXB; 0.1, 1 and 10 nM) on swine granulosa cell estradiol 17β (E2) and progesterone (P4) production (panels (**A**) and (**B**), respectively) evaluated by ELISA tests. Data, expressed as ng/mL, represent the mean ± SEM of six replicates/treatment repeated in five different experiments.

Steroidogenic activity, evaluated by ELISA assays, was not influenced by the peptide (Figure 3). 

**Figure 4:**
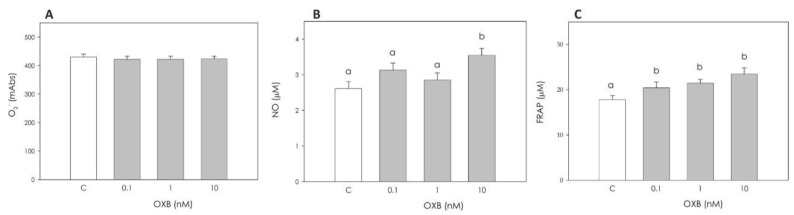
Effect of the 48 h treatment with orexin B (OXB; 0.1, 1 and 10 nM) on swine granulosa cell superoxide anion generation (O_2_^−^) using WST assay (**A**), nitric oxide (NO) production using Griess assay (**B**) and non-enzymatic scavenging activity using FRAP assay (**C**). Data, expressed as milliAbsorbance (mAbs), represent the mean ± SEM of six replicates/treatment repeated in five different experiments. In each panel, different letters on the bars indicate a significant difference (*p* < 0.05) among treatments as calculated by ANOVA and Scheffè’s F test.

With regard to the granulosa cells’ redox status, OXB did not affect O_2_^−^ production (Figure 4A). On the contrary, NO production was significantly stimulated (*p* < 0.05) by 10 nM OXB (Figure 4B), and granulosa cell non-enzymatic scavenging activity was increased by treatment with all concentrations tested (*p* < 0.01) (Figure 4C).

### 3.2. PPO Expression and OXB Effects on Swine Aortic Endothelial Cells 

**Figure 5:**
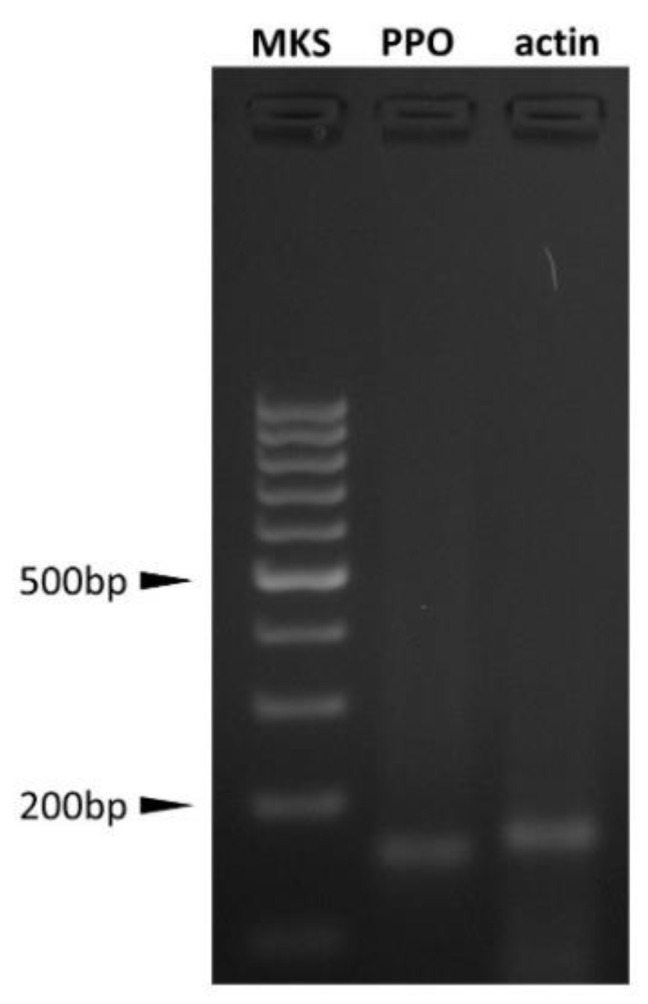
Prepro-orexin (PPO) and actin (ACT) expression in swine aortic endothelial cells (AOC). PPO expression was evaluated by nested PCR; the presence of PPO amplicon demonstrates gene expression but does not provide any information in comparison to ACT expression.

Figure 5 shows PPO expression in AOC.

**Figure 6:**
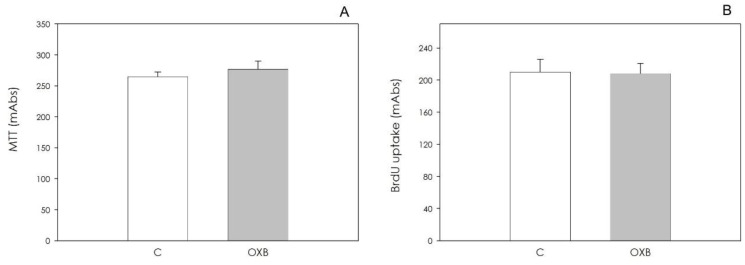
Effect of the 48 h treatment with orexin B (OXB; 10 nM) on swine aortic endothelial cells’ viability, evaluated by MTT test (**A**) and on granulosa cell proliferation evaluated using 5-bromo-2′-deoxyuridine (BrdU) incorporation assay test (**B**).

Neither cell viability nor cell proliferation were influenced by OXB (Figure 6).

**Figure 7:**
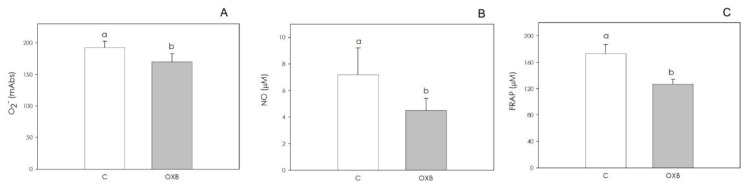
Effect of the 48 h treatment with orexin B (OXB; 10 nM) on swine aortic endothelial cells’ superoxide anion generation (O_2_^−^) using WST assay (**A**), nitric oxide (NO) production using Griess assay (**B**) and non-enzymatic scavenging activity using FRAP assay (**C**). Data, expressed as milliAbsorbance (mAbs), represent the mean ± SEM of six replicates/treatment repeated in five different experiments. In each panel, different letters on the bars indicate a significant difference (*p* < 0.05) among treatments as calculated by ANOVA and Scheffè’s F test.

On the contrary, redox status was affected by the peptide, which significantly inhibited (*p* < 0.05) both O_2_ and NO generation (Figure 7A,B). Non-enzymatic scavenging activity was also significantly (*p* < 0.05) reduced (Figure 7C).

**Figure 8:**
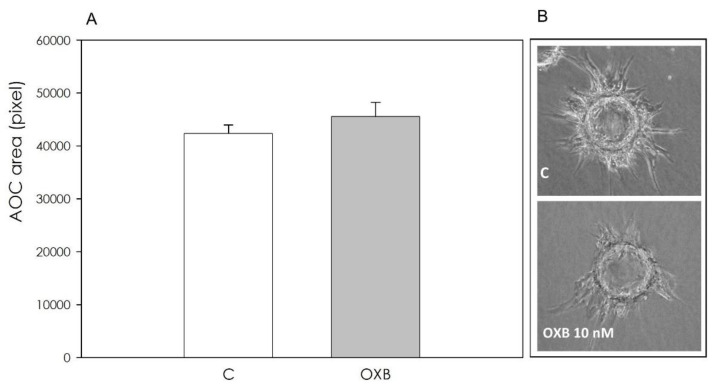
Effect of the 48 h treatment with OXB (10 nM) on aortic endothelial cell (AOC) growth in fibrin gels. Panel (**A**): data, expressed as pixels, represent the mean ± SEM of six replicates/treatment repeated in five different experiments. Panel (**B**): exemplary images of the AOC growing on collagen-coated MC beads in fibrin gels, both after 48 h of treatment with OXB (10 nM).

**Figure 9:**
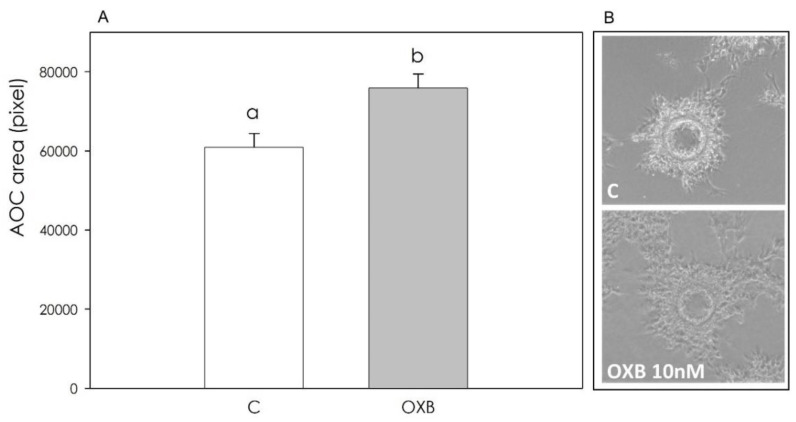
Effect of the 72 h treatment with OXB (10 nM) on aortic endothelial cell (AOC) growth in fibrin gels. Panel (**A**): data, expressed as pixels, represent the mean ± SEM of six replicates/treatment repeated in five different experiments. Different letters on the bars indicate a significant difference (*p* < 0.001) among treatments as calculated by ANOVA and Scheffè’s F test. Panel (**B**): exemplary images of the AOC growing on collagen-coated MC beads in fibrin gels both after 72 h of treatment with OXB (10 nM).

Fibrin gel angiogenesis bioassay revealed that OXB, while ineffective after a 48 h treatment (Figure 8), exerted a significant (*p* < 0.001) stimulatory effect after 72 h (Figure 9).

## 4. Discussion

The involvement of OXA and OXB in the modulation of reproductive activity is supported by different studies documenting their regulatory role on the HPG axis [13] by means of a modulation of GnRH and LH secretion in different species [21,39,40,41]. In addition, orexins appear also to be involved in the local control of gonadal physiology. Our research group has previously demonstrated the presence and expression of the orexinergic complex in the pig ovary [15,16,17]. In particular, we have shown that OXA and its receptors are expressed both in the corpus luteum as well as in granulosa cells. The physiological role played by OXA in the ovary is further supported by our results documenting its effects on both luteal and granulosa cell functions [15,16,17].

The present data document the fact that OXB is immunolocalized in both swine granulosa and thecal cells and is a likely candidate to play a role in the local regulation of reproductive activity. Our data show that the examined peptide does not affect granulosa cell growth and steroidogenesis. A previous report [12] is only partially in accordance with our finding, since it demonstrates an inhibitory effect induced by OXB on estradiol output. However, it should be noted that the cell model used in the study is relative to granulosa cells which underwent luteinization due to the presence of serum in culture medium. The effect of OXB was also tested in the presence of FSH, while we explored the effect of OXB in the absence of the gonadotropin, to study its potential influence in basal conditions as in our previous studies [7,17]. Further studies should be planned to investigate the steroidogenic effect of OXB in the presence of FSH stimulation. Moreover, it should be noted that the orexin peptides bind selectively to the OX1 and OX2 receptors (OX1R and OX2R, also known as HCRTR1 and HCRTR2). These are G-protein-coupled receptors that have seven transmembrane domains and some similarity to other neuropeptide receptors. OX1R and OX2R are strongly conserved across mammals, with 94% identity in the amino acid sequences between humans and rats. OX1R binds OXA with high affinity (IC50 20 nM in a competitive binding assay), but it has considerably less affinity for OXB (IC50 420 nM) [42]. These aspects could be involved and the effects of receptor antagonists need to be investigated in further experiments. However, even though most granulosa cell functions were not modified by OXB treatment, including the generation of superoxide anion, significant effects on redox status were observed, namely an increase in both nitric oxide and non-enzymatic scavenger activities. As a general remark, the effect of OXB has been less investigated, possibly due to its considerably lesser affinity for OX2. However, Sokołowska et al. [43] demonstrated a potent antioxidant effect of OXB in primary neuronal rat culture and a therapeutic use has also been hypothesized [44]. It should be noted that our findings are of particular interest, since redox status regulation generally plays a central role in follicular function. In particular, a crucial modulatory action on ovarian physiology has been demonstrated for a multifaced molecule such as nitric oxide [45].

Potential effects of OXB on endothelial cell redox status and nitric oxide secretion were also studied in swine aortic cells; our data document the fact that OXB can also affect endothelial cell free radical balance, since both superoxide anion and nitric oxide production appeared reduced, as well as non-enzymatic scavenging activity. A protective effect against oxidative stress has already been demonstrated for OXA [46]. To our knowledge, these aspects have never been investigated before, but deserve to be further explored, since redox status is crucial for a physiological endothelial function [47]. Our data point out a local expression of OXB, thus suggesting its potential involvement in the regulation of cellular function. PPO expression has been previously assessed in rat aortic cells by Johren et al. [48] and several studies [49,50] indicate effects of orexins in cardiovascular functions. It has been previously shown [51] that OXA induces angiogenesis via the orexin receptors, indicating that the peptide and its receptors play important roles in new vessel growth under pathophysiological conditions. To our knowledge, the effect of OXB has never been investigated before. Our data indicate that, while OXB appeared ineffective in modulating endothelial cells’ growth, it displayed a stimulatory effect on angiogenesis, as evaluated by means of our in vitro three-dimensional fibrin gel bioassay. This result would suggest a fundamental role offered by the fibrin mesh, a three-dimensional (3D) support on which endothelial cells organize into capillary-like structures on the surface of the substrate and invade the surrounding matrix, developing tubular structures inside the matrix. The three-dimensional model reflects more closely the in vivo situation. The three-dimensional assay supports capillary invasion into the matrix, outgrowth of tubular structures, sprouting from cells attached on microcarrier beads, and formation of capillary networks. Overall, these aspects need to be better clarified since angiogenesis plays a pivotal role in follicular growth and development [52].

## 5. Conclusions

The present study expands our previous findings on the orexin system in swine ovarian follicles, demonstrating a potential functional role of OXB in the control of redox status in granulosa and endothelial cells and suggesting an involvement of the peptide in the follicular angiogenic events.

## Data Availability

Data are available upon request.
